# Efficient charge separation and visible-light response in bilayer HfS_2_-based van der Waals heterostructures

**DOI:** 10.1039/c8ra03047b

**Published:** 2018-05-23

**Authors:** Biao Wang, Xukai Luo, Junli Chang, Xiaorui Chen, Hongkuan Yuan, Hong Chen

**Affiliations:** School of Physical Science and Technology, Southwest University Chongqing 400715 People’s Republic of China chenh@swu.edu.cn; Key Laboratory of Luminescent and Real-Time Analytical Chemistry, Ministry of Education, College of Chemistry and Chemical Engineering, Southwest University Chongqing 400715 People’s Republic of China

## Abstract

Two-dimensional (2D) hafnium disulfide (HfS_2_) has been synthesized and is expected to be a promising candidate for photovoltaic applications, and at the same time the hexagonal BN sheet (h-BN) and graphene-like C_3_N_4_ sheet (g-C_3_N_4_) have also been fabricated and are expected to be applied in photocatalysis. In this work, we employ hybrid density functional theory to investigate HfS_2_-based van der Waals (vdW) heterojunctions for highly efficient photovoltaic and photocatalytic applications. HfS_2_/h-BN and HfS_2_/g-C_3_N_4_ heterostructures with direct bandgaps and efficient charge separation are both typical type-II semiconductors and have potential as photovoltaic structures for solar power. Moreover, compared with h-BN and g-C_3_N_4_ single-layers, HfS_2_/h-BN heterostructures with 6% tensile strain and HfS_2_/g-C_3_N_4_ heterostructures with 9% tensile strain have moderate bandgaps, whose optical absorption is obviously enhanced in the ultraviolet-visible (UV-VIS) light range and whose bandedges are suitable for photocatalytic water splitting. HfS_2_/h-BN heterostructures with 6% applied strain, being different from HfS_2_/g-C_3_N_4_ heterostructures with 9% strain, possess a direct bandgap and show complete separation of the photoinduced electron–hole pairs. Thus the HfS_2_/h-BN heterojunction with 6% strain has bright prospects for use in visible light photocatalytic water splitting to produce hydrogen.

## Abstract 2

In this work, we employ hybrid density functional theory to investigate HfS_2_-based van der Waals (vdW) heterojunctions for highly efficient photovoltaic and photocatalytic applications.

## Graphical abstract

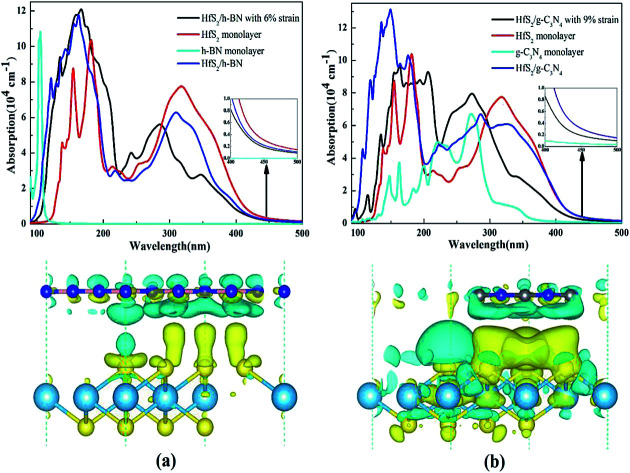

## 1 Introduction

Since the discovery of graphene,^1^ ultrathin 2D nanomaterials^2,3^ have attracted widespread attention due to their peculiar properties and high utilization efficiency of specific surface areas, which are of significance for potential applications such as photocatalysis. Attributed to the steady improvements made in laboratories recently, many types of monolayer or few-layer transition metal dichalcogenides (TMDCs;^4^*e.g.*WS_2_,^5^ MoS_2_,^6^ HfS_2_,^7^ ZrS_2_,^8^*etc.*) have been successfully fabricated and have become the focus of researchers.

Because HfS_2_ is predominantly an ionic crystal with a moderate bandgap and a “two-dimensional” layered structure bonded by weak van der Waals forces,^9^ it has been inspiring researchers to study its properties from bulk to monolayer.^10–12^ Recently, few-layered hafnium disulfide nanosheets with a 1T-structure have been successfully made by chemical vapor deposition, which shows their bright prospects in the fields of photodetectors^7^ and field effect transistors^13^ due to their ultrafast photoresponse time, high photosensitivity and excellent field effect responses. Furthermore, theoretical calculations suggest that the monolayers of 1H- and 1T-HfS_2_ have potential applications as photocatalysts for water splitting.^14^ However, individual HfS_2_ nanosheets similar to graphitic carbon nitride (g-C_3_N_4_)^15^ are liable to rapidly recombine photogenerated electron–hole pairs and reduce solar conversion and photocatalytic efficiency.

For the sake of reducing bandgaps and enhancing the separation of the photoinduced electron–hole pairs,^16^ a number of vdW heterojunctions composed of different single-layer materials that are combined together by vdW forces have been manufactured and have been predicted to acquire more desirable properties.^2,8,17–24^ At present, 2D HfS_2_/phosphorene heterojunctions^25^ as field effect transistors and HfS_2_-based nanocomposites as Z-scheme type photocatalysts for hydrogen production^26^ have been studied, whose structures and photocatalytic mechanism are more complex than those of type-II heterojunctions. Because of the high cost and difficulty in manufacturing 2D heterojunctions in the laboratory, theoretical research is necessary to predict whether or not they can be used as catalysts for hydrogen evolution by water splitting.^26^ Moreover, the g-C_3_N_4_ and hexagonal boron nitride (h-BN) monolayers have become elementary slabs for vdW heterostructures.^27^ Here, we mainly investigate whether the bilayer vdW heterostructures with combined HfS_2_ and h-BN (g-C_3_N_4_) layers can form standard type-II heterojunctions, which are deemed to be able to enhance the separation of electron–hole pairs. In addition, their band gaps and band edge positions, the effect of the biaxial strain, the density of states and optical absorption spectra are calculated by density functional theory.

## 2 Computational method

Our first-principles calculations are carried out using the Vienna ab initio simulation package (VASP)^28,29^ with the projector augmented wave (PAW) method.^30^ Electronic exchange and correlation effects are described by the generalized gradient approximation (GGA)^31^ in the parametrization of Perdew–Burke–Ernzerhof (PBE).^32^ Since it is well known that PBE generally underestimates the bandgaps of semiconductors, the Heyd–Scuseria–Ernzerhof hybrid functional (HSE06),^33^ which is considered a more precise and widely applied method, has been chosen to calculate their electronic and optical properties. The vdW interactions are taken into account in these heterojunctions by employing Grimme’s DFT-D3 method.^34^ A default mixing parameter (*α* = 0.25) is adopted to compute the exchange correlation energy in our work. In the following computations, a Monkhorst–Pack mesh^35^ for k-point sampling is set as 7 × 7 × 1. A cutoff energy of 550 eV is chosen. The convergence criteria of the geometry optimizations are set to less than 10^−5^ eV for the total energy and 0.01 eV Å^−1^ for the force on each ion. A vacuum space of 20 Å is inserted to avoid the interactions between adjacent heterojunctions. The vacuum space is used to evaluate the absolute position of the band compared with that of the standard hydrogen electrode potential. Therefore, the absolute bandedge positions are computed *via* subtracting the vacuum level by means of averaging the LOCPOT file.

## 3 Results and discussion

### 3.1 Structural stability

**Fig. 1:**
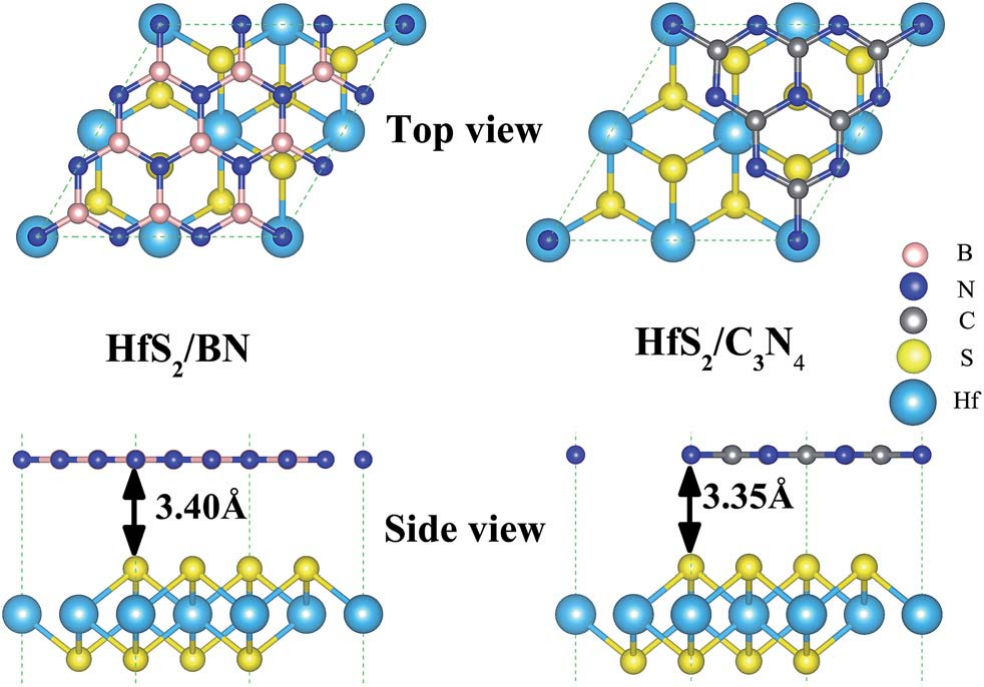
The optimized geometry of the vdW heterojunctions. The pink, blue, gray, yellow and sky blue balls indicate B, N, C, S and Hf atoms, respectively. Top: top view of the heterojunctions, bottom: side view of the heterojunctions. Left: HfS_2_/h-BN heterojunction, right: HfS_2_/g-C_3_N_4_ heterojunction.

HfS_2_ monolayers in a 1T-phase have been theoretically predicted to be globally stable.^14^ Moreover, 1T-phase few-layered HfS_2_ nanosheets have been successfully fabricated in experiments.^7^ Therefore, we adopt the 1T-phase of 2D HfS_2_ in the following calculations. Our calculated optimized lattice constant of the HfS_2_ monolayer is 3.61 Å, which matches well with the previous experimental value of 3.62 Å.^10^ As shown in Fig. 1, the geometric configurations of these heterojunctions are composed of a 2 × 2 HfS_2_ (*a* = 3.61 Å) single-layer supercell, and a unit cell of g-C_3_N_4_ (*a* = 7.13 Å)^36^ nanosheet or a 3 × 3 h-BN (*a* = 2.50 Å)^37^ single-layer supercell (Fig. 1). There exists lattice mismatch, which is illustrated by this formula: *ε* = (*l*_others_ − *l*_HfS_2__)/*l*_HfS_2__, where *l*_others_ represents the unit cell of the g-C_3_N_4_ single-layer (*a* = 7.13 Å) and the 3 × 3 h-BN supercell (*a* = 7.50 Å), and *l*_HfS_2__ corresponds to the 2 × 2 HfS_2_ supercell (*a* = 7.22 Å). As listed in Table 1, the lattice mismatches are −1.16% and 3.89% for g-C_3_N_4_ and h-BN, which indicates that the HfS_2_ nanosheet meets the geometric lattice-matching demand to form the vdW heterojunctions.

**Table 1:** The geometric parameters, binding energies and work functions of the heterojunctions

Structure	*ε* (%)	*R* (Å)	*E*_b_ (eV)	*Φ* (eV)
HfS_2_/h-BN	3.89	3.40	−0.11	6.09
HfS_2_/g-C_3_N_4_	−1.16	3.35	−0.58	6.74

As shown in Fig. 1, the equilibrium distances of the fully relaxed nanocomposites, marked as *R* in Table 1, are 3.40 Å and 3.35 Å for HfS_2_/h-BN and HfS_2_/g-C_3_N_4_ heterojunctions, respectively, and represent the space between the S atom in the HfS_2_ layer and the other layer. The equilibrium distances of the studied heterostructures are close to those of other vdW heterostructures,^8,24^ which are typical distances of vdW heterostructures. Therefore, the vdW correction of interactions has been applied when optimizing the structure and calculations. The binding energy (*E*_b_) is defined by the equation: *E*_b_ = *E*_x/HfS_2__ – (*E*_x_ + *E*_HfS_2__), where *E*_x/HfS_2__, *E*_x_ and *E*_HfS_2__ represent the total energy of the nanocomposites, the independent single-layers (x = h-BN or g-C_3_N_4_) and the isolated HfS_2_ monolayer, respectively. The computed binding energies of the HfS_2_/h-BN and HfS_2_/g-C_3_N_4_ heterojunctions (Table 1) are −0.11 eV and −0.58 eV respectively, which means that these nanocomposites are stable.

### 3.2 Band structure and density of states

**Fig. 2:**
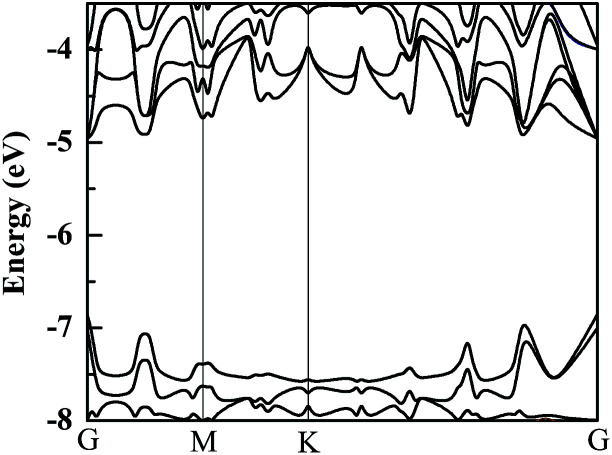
The band structure of a HfS_2_ monolayer with SOC correction.

Because Hf is a heavy element, the spin-orbital coupling (SOC) correction has been investigated in previous research and it was discovered that the SOC effect in the HfS_2_ single-layer is small.^26^ The band structure of a HfS_2_ monolayer with SOC correction is calculated and presented in Fig. 2. Furthermore, we examine the effect of SOC on the HfS_2_ monolayer and related heterojunctions, and find that its influences on their bandgaps are all less than 0.1 eV. Therefore, in consideration of the heavy price of calculations, the SOC correction isn’t employed in the following calculations. Using the HSE06 functional, the calculated bandgap of the HfS_2_ monolayer is 1.99 eV, which approximates to the previous computed value of 2.06 eV.^12,26^ The bandedge of the HfS_2_ monolayer can’t straddle the water reduction potential shown in Fig. 2, which means that the HfS_2_ single-layer isn’t able to be directly used for photocatalytic hydrogen production from water splitting. The calculated bandgaps of the g-C_3_N_4_ and h-BN monolayers are 2.76 eV and 5.69 eV, respectively. Moreover, the catalytic activity depends on the related work function (*Φ*), which represents the minimum quantity of work required to remove an electron from the interior of materials to the vacuum level. Work function is defined by this equation: *Φ* = *E*_v_ − *E*_F_, where *E*_v_ and *E*_F_ refer to the vacuum energy and the Fermi energy. The calculated values of the work function for HfS_2_/h-BN and HfS_2_/g-C_3_N_4_ heterojunctions are 6.09 and 6.74 eV, respectively. The values of the work function and the photocatalytic activity have an inverse relationship,^14^ which means that the catalytic activity of the HfS_2_/h-BN heterostructure is better than that of the HfS_2_/g-C_3_N_4_ heterojunction.

**Fig. 3:**
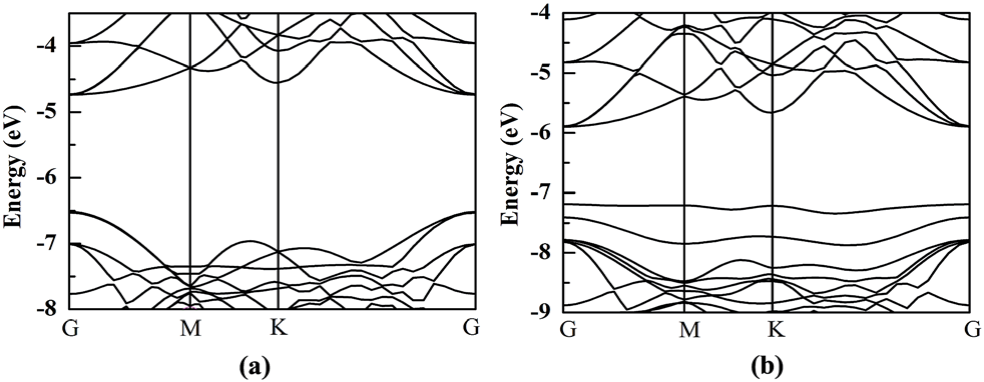
Band structures of HfS_2_/h-BN (a) and HfS_2_/g-C_3_N_4_ (b) heterojunctions.

**Fig. 4:**
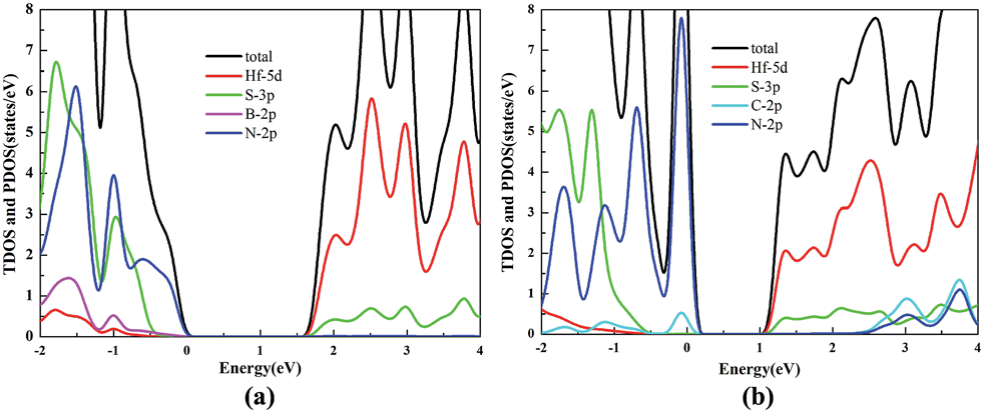
TDOS and PDOS for HfS_2_/h-BN (a) and HfS_2_/g-C_3_N_4_ (b) heterojunctions.

HfS_2_/h-BN and HfS_2_/g-C_3_N_4_ heterostructures are both direct bandgap semiconductors, whose bandgaps have been observed to reduce to 1.78 eV and 1.29 eV, as presented in Table 2. Because of these suitable bandgaps which are less than 1.8 eV, these heterostructures can effectively take advantage of solar power. It’s well known that the redox potentials of water are connected to the pH value.^38^ The reduction potential for H^+^/H_2_ is *E*_H^+^/H_2__ = −4.44 eV + pH × 0.059 eV, and the oxidation potential for O_2_/H_2_O is *E*_O_2_/H_2_O_ = −5.67 eV + pH × 0.059 eV. As shown in Table 2, *E*_g_, *E*_VBM_ and *E*_CBM_ denote the bandgap and the energy levels of the valence band maximum (VBM) and the conduction band minimum (CBM) compared to those of the vacuum level, respectively. Unfortunately, the CBMs of these heterojunctions shown in Fig. 3 aren’t higher than the reduction potential of water at pH = 0, which indicates that these heterostructures may not be used for water splitting to produce hydrogen. However, the light-generated electrons and holes are completely separated in HfS_2_/h-BN and HfS_2_/g-C_3_N_4_ heterostructures. Fig. 4 shows the total density of states (TDOS) and partial density of states (PDOS). The CB in the HfS_2_/h-BN heterojunction is composed of Hf 5d and S 3p states that originate from the HfS_2_ slab, while the VB of the heterostructure largely consists of the N 2p state which stems from the h-BN layer. Thus, the CB and VB in the heterojunction are seated in two different slabs. Similarly, the CB in HfS_2_/g-C_3_N_4_ is composed of Hf 5d and S 3p states located in the HfS_2_ layer, while the VB is composed of C 2p and N 2p states located in the other layer. Therefore, both of the heterostructures form typical type-II semiconductors, which can improve the efficiency of utilizing sunlight. With moderate bandgaps and full separation of photogenerated electron–hole pairs, HfS_2_/h-BN (g-C_3_N_4_) heterostructures have profound development potential as photovoltaic materials for solar power, except for in water splitting.

**Table 2:** Bandgaps and bandedges of the heterojunctions at pH = 0

Structure	*E*_g_ (eV)	*E*_VBM_ (eV)	*E*_CBM_ (eV)	Bandgap	Straddling water redox potentials
HfS_2_/h-BN	1.78	−6.51	−4.74	Direct	No
HfS_2_/h-BN with −6% strain	0.62	−5.77	−5.14	Direct	No
HfS_2_/h-BN with −3% strain	1.27	−6.21	−4.94	Direct	No
HfS_2_/h-BN with 3% strain	2.03	−6.63	−4.60	Direct	No
HfS_2_/h-BN with 6% strain	2.28	−6.71	−4.43	Direct	Yes
HfS_2_/h-BN with 9% strain	2.52	−6.80	−4.28	Direct	Yes
HfS_2_/g-C_3_N_4_	1.29	−7.19	−5.90	Direct	No
HfS_2_/g-C_3_N_4_ with −6% strain	0.01	−5.34	−5.33	Direct	No
HfS_2_/g-C_3_N_4_ with −3% strain	0.38	−5.60	−5.22	Direct	No
HfS_2_/g-C_3_N_4_ with 3% strain	1.92	−6.65	−4.73	Indirect	No
HfS_2_/g-C_3_N_4_ with 6% strain	2.29	−6.88	−4.59	Direct	No
HfS_2_/g-C_3_N_4_ with 9% strain	2.72	−7.15	−4.43	Indirect	Yes

**Fig. 5:**
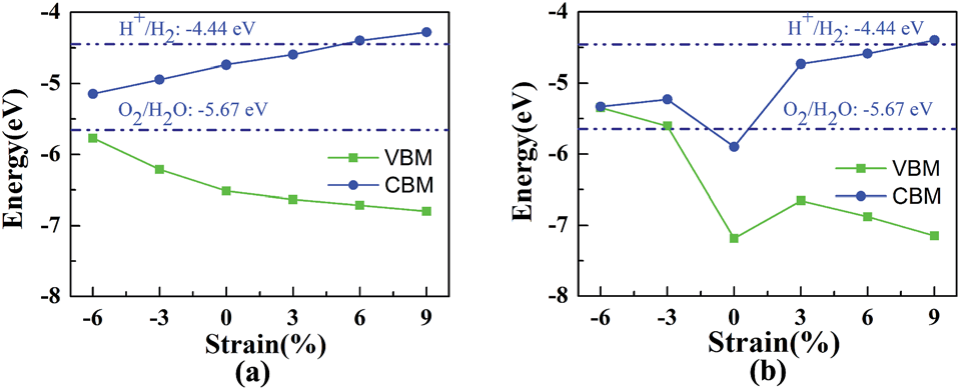
The bandedge positions of the HfS_2_/h-BN heterojunction (a) and HfS_2_/g-C_3_N_4_ heterojunction (b) as a function of biaxial strain.

In previous studies it was confirmed that the in-plane biaxial strains can effectively adjust the optical absorption and electronic properties of vdW heterostructures.^8,24^ In order to explore their potential application in the field of photocatalytic water splitting, HfS_2_/h-BN and HfS_2_/g-C_3_N_4_ heterostructures with biaxial strains have been comprehensively investigated. During relaxation of the geometric structure, the lattice constant *l*_x_ is fixed to imitate the strain on the heterostructures. Strains are defined as follows: *η* = (*l*_x_ − *l*_0_)/*l*_0_, where *l*_0_ and *l*_x_ represent the lattice of the original heterostructures and strained heterojunctions. Employing the in-plane biaxial compressive and tensile strain on these nanocomposites, the value of *η* ranges from −6% to 9%, as shown in Fig. 5, where the bandedge positions are presented as a function of the applied strains. With increasing tensile strains, the lattices of the heterostructures become larger, which indicates that the distance of the nearest atoms is enlarged. On the basis of tight-binding theory,^39^ their energy-bands will narrow down and the bandgaps will get bigger. Consequently, the enlargement of the bandgaps is accompanied by an increase in the biaxial strain as shown in Fig. 5. Due to their porosity in the g-C_3_N_4_ layer,^40^ the bandedge variation with strain in the HfS_2_/g-C_3_N_4_ heterojunction is different from that in the HfS_2_/h-BN heterojunction. Applying 6% or 9% tensile strain, the bandedge of the HfS_2_/h-BN heterostructure can straddle water redox potentials. Similarly, that of the HfS_2_/g-C_3_N_4_ heterostructure with 9% tensile strain can straddle water redox potentials as well. However, HfS_2_/g-C_3_N_4_ with increasing pressure, whose bandgap approaches to zero, can transform from semiconductor to metal. With the aim of meeting the requirements for photocatalytic water splitting, a HfS_2_/h-BN heterostructure with 6% strain and HfS_2_/g-C_3_N_4_ heterostructure with 9% strain were investigated using the following calculations.

**Fig. 6:**
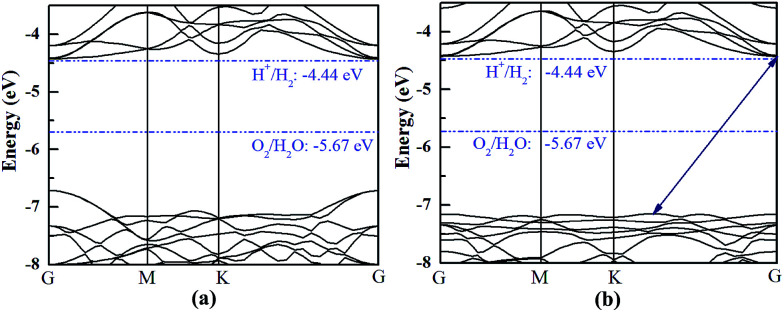
The band structures of the HfS_2_/h-BN heterostructure with 6% strain (a) and HfS_2_/g-C_3_N_4_ heterostructure with 9% strain (b) compared to the vacuum level.

The HfS_2_/h-BN heterojunction with 6% applied tensile strain is a direct bandgap semiconductor, whose bandgap is calculated to be 2.28 eV. The VBM and CBM of the heterojunction are both at the G point, as plotted in Fig. 6. Meanwhile the HfS_2_/g-C_3_N_4_ heterostructure with 9% applied tensile strain, whose VBM is at the G point and CBM is between the G and K points, is an indirect bandgap semiconductor with a bandgap of about 2.72 eV. Considering the moderate and direct bandgap, the photocatalytic performance of the HfS_2_/h-BN heterojunction with 6% strain is superior to that of the other heterostructures.

**Fig. 7:**
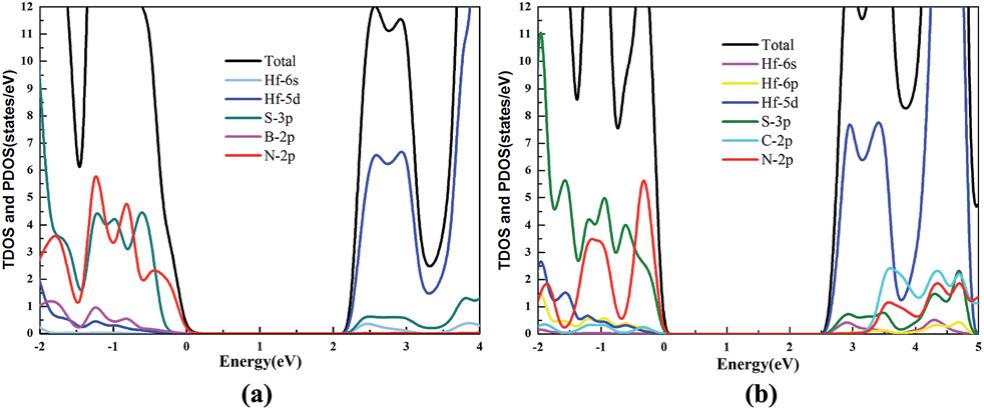
The TDOS and PDOS for the HfS_2_/h-BN heterostructure with 6% strain (a) and HfS_2_/g-C_3_N_4_ heterostructure with 9% strain (b).

The TDOS and PDOS of the HfS_2_/h-BN heterostructure with 6% strain and HfS_2_/g-C_3_N_4_ heterostructure with 9% strain are presented in Fig. 7. The CB of the HfS_2_/h-BN heterostructure with 6% strain is mainly composed of Hf 5d, Hf 6s and S 3p orbitals derived from the HfS_2_ layer, while the VB of the heterojunction is primarily composed of the N 2p state stemming from the h-BN layer. Obviously, this heterojunction is a classic type-II semiconductor, where the CB and VB are located on different layers in favor of complete separation of the electron–hole pairs and separate production of hydrogen and oxygen. For the HfS_2_/g-C_3_N_4_ heterostructure with 9% strain, the VB of the heterojunction is composed of N 2p and S 3p orbitals that originate from both layers, and the CB is composed of the Hf 5d, Hf 6s and S 3p orbitals. As a result, the HfS_2_/g-C_3_N_4_ heterostructure with 9% strain is incapable of completely separating the photoinduced carriers. Therefore, considering the efficient division of the electron–hole pairs, the HfS_2_/h-BN heterostructure with 6% strain is more suitable for photocatalysis than the HfS_2_/g-C_3_N_4_ heterostructure with 9% strain.

### 3.3 Charge transfer analysis

**Fig. 8:**
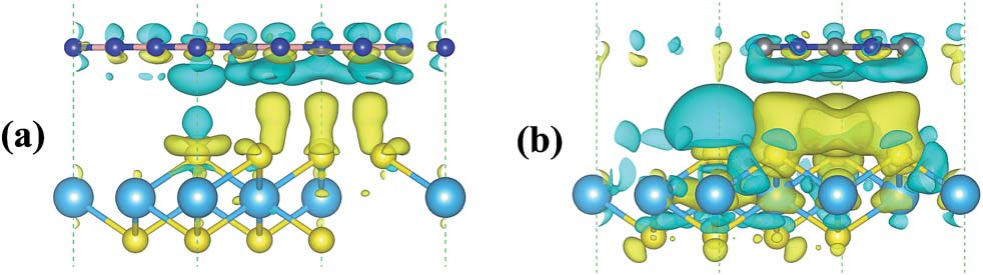
3D charge density differences in the HfS_2_/h-BN heterojunction with 6% applied strain (a) and HfS_2_/g-C_3_N_4_ heterojunction with 9% applied strain (b) with an isovalue of 0.00012 e Å^−3^. The yellow and sea-green isosurfaces represent charge aggregation and depletion, respectively.

Since strengthened photocatalytic performance arises from the effective separation and transfer of the photogenerated carriers,^41^ the three dimensional (3D) charge density differences at the interfaces of the heterojunctions are depicted in Fig. 8. This can be calculated using the following equation: *ρ*_dif_ = *ρ*_HfS_2_/Y_ − *ρ*_HfS_2__ − *ρ*_Y_, where Y and HfS_2_/Y denote h-BN or g-C_3_N_4_ and HfS_2_-based heterojunctions, respectively. As shown in the figure, a great deal of electrons are redistributed in the interfaces of the heterojunctions, which leads to built-in electric fields and facilitates separation of the photogenerated electron–hole pairs. For the sake of quantitative analysis of the charge variation at these interfaces, Bader charge analysis^42^ has been applied to these heterojunctions. Based on the calculations, the Hf, B and C atoms are inclined to deplete electrons, while the S and N atoms are inclined to aggregate electrons. Moreover, the calculated quantity of the charge transfer from the h-BN and g-C_3_N_4_ layer (the top layer in Fig. 8) to the HfS_2_/layer (the bottom layer in Fig. 8) is 0.0056 *e* and 0.0165 *e*, respectively. The calculated quantity of the charge transfer is in the same order of magnitude as that for typical vdW heterostructures.^43^ Obviously, a great deal of charge accumulation is observed in the interface of the lower layer shown in Fig. 8, while the charge depletion appears on the upper layer. Therefore, our work reveals that these HfS_2_-based heterojunctions can effectively enhance the separation and transfer of electron–hole pairs and then improve the photocatalytic performance.

### 3.4 Optical properties

**Fig. 9:**
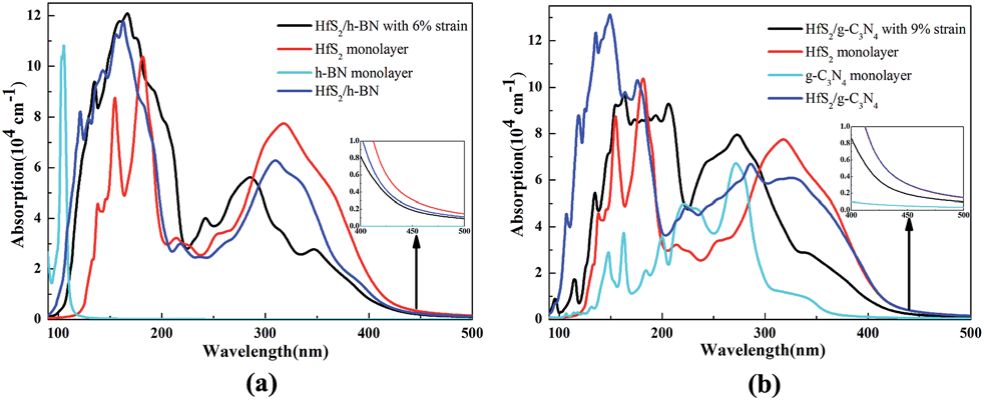
Optical properties of HfS_2_/h-BN heterojunctions (a) and HfS_2_/g-C_3_N_4_ heterojunctions (b).

The calculated optical absorption spectra of the g-C_3_N_4_, h-BN and HfS_2_ single-layers and related heterojunctions as a function of wavelength are illustrated in Fig. 9. Compared with the optical absorption of the h-BN and g-C_3_N_4_ monolayer, the optical absorption intensity of the corresponding HfS_2_-based heterojunctions is dramatically strengthened in the ultraviolet-visible (UV-VIS) light region, which is in accordance with reduction of the band gap. Whether the HfS_2_/h-BN heterostructures have applied strain or not, the optical absorption edges all shift to the visible light range (more than 500 nm), as plotted in Fig. 9.

Moreover, the optical absorption spectra of the HfS_2_/h-BN heterojunction (with 6% strain) depicted in Fig. 9(a) are obviously enhanced in the ultraviolet range in contrast to those of the HfS_2_ single-layer. Compared with the optical absorption spectrum of the h-BN monolayer, it can only be speculated that the significant red shift of the HfS_2_/h-BN heterojunction’s absorption spectrum (with 6% strain) is mainly induced by a transition from the N 2p to Hf 5d orbital, as shown in Fig. 5(a) (Fig. 7(a)), which is important for strengthening the heterojunction’s photocatalytic activity. Consequently, with an ideal band gap, complete charge separation and visible-light response, the HfS_2_/h-BN heterojunction can be used in the field of photovoltaics. On account of its moderate bandgap, well positioned band edge, effective charge separation and reinforced UV-VIS light absorption, the HfS_2_/h-BN heterojunction with 6% strain can be used for photocatalytic water splitting.

As shown in Fig. 9(b), the optical absorption edge of the HfS_2_/g-C_3_N_4_ heterojunction (with 9% strain) shifts to a longer wavelength range. In particular, the HfS_2_/g-C_3_N_4_ heterojunction takes full advantage of visible light, which is similarly derived from a transition from the N 2p to Hf 5d orbital, as shown in Fig. 5(b). Because of its relatively narrow bandgap, efficient charge separation and visible-light response, the HfS_2_/g-C_3_N_4_ heterojunction is a promising material for photovoltaic applications. With an indirect and moderate bandgap, the HfS_2_/g-C_3_N_4_ heterojunction with 9% applied strain has a well positioned band edge but isn’t able to completely separate charge carriers. Therefore, the photocatalytic performance of the HfS_2_/h-BN heterojunction with 6% strain is superior to that of the HfS_2_/g-C_3_N_4_ heterojunction with 9% strain.

## 4 Conclusions

In conclusion, we have systematically studied the HfS_2_/h-BN and HfS_2_/g-C_3_N_4_ heterojunctions by using the HSE06 hybrid functional. We find that these heterostructures are both direct bandgap semiconductors, whose bandgaps have been observed to reduce to 1.78 eV and 1.29 eV, respectively. The CB and VB of the heterostructures are both derived from different layers, effectively avoiding recombination of the photoinduced electron–hole pairs. Owing to an ideal band gap, complete separation of the photoinduced charge carriers and a visible-light response, these heterojunctions can be widely used in the photovoltaic field. However, the bandedges of these heterojunctions are incapable of straddling water redox potentials.

In addition, applying biaxial strain can effectively adjust the electronic properties of HfS_2_/h-BN and HfS_2_/g-C_3_N_4_ heterojunctions. With pressure of a certain extent, the bandgap of the HfS_2_/g-C_3_N_4_ heterojunction can reduce to 0 eV, which may indicate that the heterojunction transforms from semiconductor to metal. The band edges of HfS_2_/h-BN composites with 6% tensile strain and HfS_2_/g-C_3_N_4_ heterojunctions with 9% tensile strain are both able to straddle water redox potentials. The photocatalytic performance of a HfS_2_/h-BN heterojunction with 6% strain, which is a typical type-II semiconductor, has advantages over that of a HfS_2_/g-C_3_N_4_ heterojunction with 9% strain. Because of the moderate band gap, effective charge separation, well positioned band edge and reinforced UV-VIS light absorption, HfS_2_/h-BN heterojunctions with applied strains are a promising photocatalyst for water splitting. These calculations pave the way for exploiting high-performance HfS_2_-based photocatalysts.

## Conflicts of interest

There are no conflicts to declare.

## Supplementary Material
